# Iodine-Biofortified Lettuce Can Promote Mitochondrial Dependent Pathway of Apoptosis in Human Gastrointestinal Cancer Cells

**DOI:** 10.3390/ijms24129869

**Published:** 2023-06-07

**Authors:** Olga Sularz, Aneta Koronowicz, Sylwester Smoleń, Cayla Boycott, Barbara Stefanska

**Affiliations:** 1 Department of Human Nutrition and Dietetics, Faculty of Food Technology, University of Agriculture in Krakow, Balicka 122, 31-149 Krakow, Poland; sularz.olga@gmail.com; 2 Department of Plant Biology and Biotechnology, Faculty of Biotechnology and Horticulture, University of Agriculture in Krakow, Al. 29 Listopada 54, 31-425 Krakow, Poland; sylwester.smolen@urk.edu.pl; 3 Food, Nutrition and Health Program, Faculty of Land and Food Systems, The University of British Columbia, 2205 East Mall, Vancouver, BC V6T 1Z4, Canada; caylasboycott@gmail.com

**Keywords:** protein expression, apoptosis, gastrointestinal cancer, iodine

## Abstract

Previously, our research provided evidence that exposure of gastric and colon cancer cells to extracts from iodine-biofortified lettuce leads to a reduction of cell viability and proliferation through cell cycle arrest and upregulation of pro-apoptotic genes. The aim of the present study was to determine the potential cellular mechanisms of induction of cell death in human gastrointestinal cancer cell lines after treatment with iodine-biofortified lettuce. We demonstrated that extracts from lettuce enriched with iodine induce apoptosis in gastric AGS and colon HT-29 cancer cells and the mechanism of programmed cell death may be triggered and executed through different signaling pathways, depending on the type of cells. Western blot analysis revealed that iodine-fortified lettuce leads to cell death through the release of cytochrome c to the cytosolic fraction and activation of the primary drivers of apoptosis: caspase-3, caspase-7, and caspase-9. Furthermore, we have reported that apoptotic effects of lettuce extracts may be mediated by poly (ADP-ribose) polymerase (PARP) and activation of pro-apoptotic Bcl-2 family proteins such as Bad, Bax, and BID. We also observed mitochondrial dysfunction with the dissipation of the mitochondrial membrane potential in cells exposed to lettuce extracts. Taken together, these results indicate that the organic form of iodine such as 5-ISA and 3,5-diISA is an important factor in the activation of intrinsic mitochondrial apoptotic pathway in AGS and HT-29 cancer cells in a p53-independent manner.

## 1. Introduction

Combating cancer is the grand challenge faced by public health because, despite the high level of development of medicine and implementation of novel and precision therapeutics, cancer is still mentioned among the most common causes of death in the world [1]. One of the hallmarks of cancer cells, that makes it difficult to eradicate them, is their ability to evade programmed cell death by altering the expression of anti- and pro-apoptotic genes and the stability of apoptosis-related proteins [2,3]. Therefore, widely used methods in cancer treatments, including chemotherapy, immunotherapy, or radiotherapy supporting surgical procedures, are focused on the activation of apoptotic signaling pathways and induction of programmed cell death in cancer cells [4].

In recent years, there is a growing body of literature emphasizing the role of plant-derived natural products and plant extracts in support of cancer treatments via activation of apoptosis [2,5,6,7].

Consequently, a lot of attention is paid to plant polyphenols, well-known as natural antioxidants that are commonly found in fruits and vegetables and exert chemopreventive effects through multiple mechanisms [8]. The chemopreventive activity of dietary polyphenols is related to their regulatory role in signaling pathways involved in apoptosis, cell survival or proliferation, inhibition of DNA synthesis, and induction of cell cycle arrest [8]. In addition, it was shown that the induction of apoptosis in cancer cells may be related to pro-oxidant activities of polyphenols. These compounds can mediate reactive oxygen species (ROS) generation via the mitochondrial intrinsic apoptotic pathway and consequently disrupt the potential of the mitochondrial membrane resulting in its rapid depolarization [8,9,10].

Studies have proven that iodine may demonstrate anticancer activity as well via the intracellular formation of derivative 6-iodolactone (6-IL), activation of peroxisome proliferator-activated receptor (PPARγ), and disruption of the mitochondrial membrane potential [11,12,13]. Antiproliferative and apoptotic properties of iodine (I2) and/or 6-IL were observed in human cancerous breast, prostate, lung, and neuroblastoma cells [14,15,16]. It has been shown that the anticancer effects of iodine are dose-dependent in MCF-7 breast cancer cells. Iodine at low concentrations exerted apoptotic effects through binding with arachidonic acid (AA) and leading to activation of PPAR and triggering BAX/caspase-dependent apoptosis. However, iodine at higher concentrations acted as an oxidizing agent activating cell death by the AIF/PARP1 pathway without the participation of caspases [15].

Our previous research, concerning the chemical composition of lettuce biofortified with iodine compounds, showed that enriched plants have a high antioxidant potential due to an increased content of certain vitamins and polyphenols [17]. It is found that extracts from iodine-enriched lettuce have the ability to induce the generation of reactive oxygen species (ROS) in gastrointestinal cancer cell lines [18]. In addition, we showed that the lettuce extracts exert anticancer properties through the inhibition of viability and proliferation of human AGS and HT-29 cancer cells [19]. We suggested that these anticancer effects result from arresting the cell cycle and modifying the expression of genes implicated in apoptosis, cell proliferation, and oncogenic signaling pathways. Moreover, in HT-29 cells, we demonstrated the epigenetic activation of the DNA methylation-silenced SEMA3A tumor suppressor gene upon 5-ISA-fortified lettuce extract [19]. These findings prompted us to further investigate the molecular mechanism underlying the anticancer activity of iodine-fortified lettuce in AGS and HT-29 cancer cell lines.

Therefore, in this study, we performed further analysis to determine the potential mechanism of induction of apoptosis in gastric and colon cancer cells treated with extracts from iodine-fortified lettuce. We have found that the lettuce extracts led to an increase in the percentage of apoptotic and total depolarized cells in both cell lines. We observed overexpression of cleaved forms of caspases and pro-apoptotic proteins and the involvement of pathways regulating mitochondrial membrane permeability and release of cytochrome c.

## 2. Results

### 2.1. *Lettuce Extracts Induce Apoptosis in AGS and HT-29 Cells*

**Figure 1:**
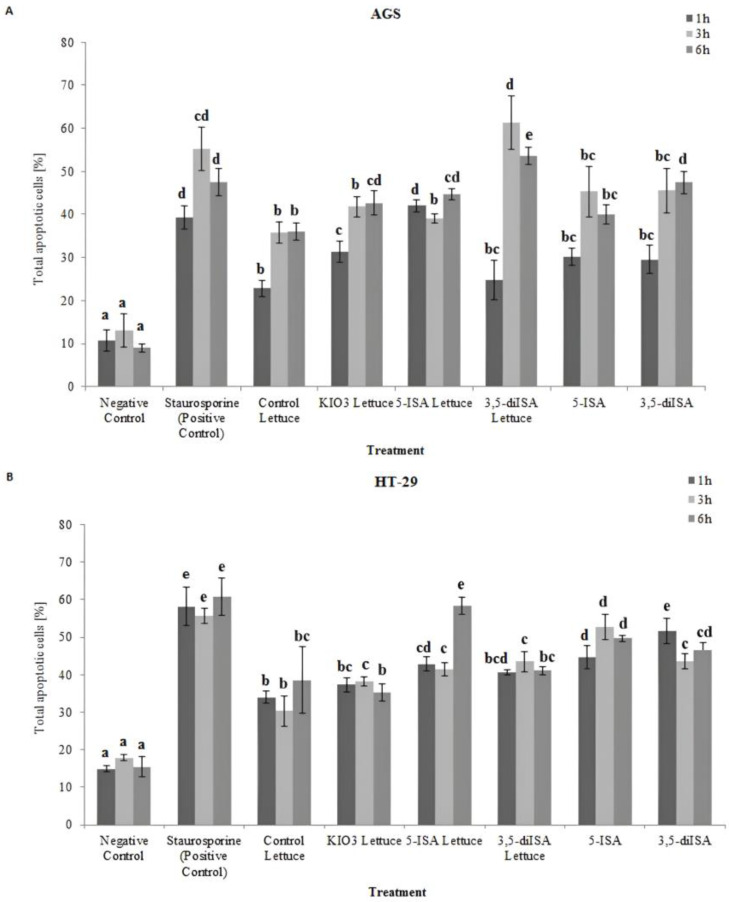
The effect of extracts from iodine-biofortified lettuce on apoptosis in human gastrointestinal cell line AGS (**A**) and colorectal adenocarcinoma cell line HT-29 (**B**). Cells were treated for 1, 3, and 6 h with 1000 µg/mL extracts from control lettuce, or KIO_3_-fortified lettuce, or 5-ISA-fortified lettuce, or 3,5-diISA-fortified lettuce, or synthetic 5-ISA, or synthetic 3,5-diISA, or 1.5 µM staurosporine as positive control. Statistical significance was assessed using one-way ANOVA followed by Tukey’s post hoc. Columns with the same letter are not significantly different (*p* > 0.05). Different letters indicate statistically significant differences at *p* < 0.05.

To determine whether extracts from iodine-biofortified lettuce and synthetic forms of iodosalicylic acids inhibit cancer cell growth and proliferation through induction of apoptosis, the Muse™ Annexin V & Dead Cell Assay (Merck Millipore, Billerica, MA, USA) was performed (Figure 1 and representative plots in Supplementary Figure S1). Cancer cells were treated with lettuce extracts and synthetic iodosalicylates for 1, 3, and 6 h and apoptotic cells were detected by Annexin V/7-AAD staining. As we showed in our previous study [19], the concentration of biofortified lettuce was chosen on the basis of the dose–response relationship. The 1000 µg/mL concentration that was used for the presented analyses induced the highest decrease in cell viability with simultaneously the lowest cytotoxicity level to avoid necrosis effect in cancer and normal cells [19].

In AGS gastric cancer cells as well as HT-29 colon cancer cells, treatments with extracts from lettuce fortified with iodine caused a profound increase in the population of total apoptotic cells (including early and late apoptotic cells), as compared to the negative control. In AGS cells, treatment with 1000 µg/mL extracts from lettuce induced a statistically significant increase from about 40% of total apoptotic cells in control lettuce to about 60% when treating with 3,5-diISA-fortified lettuce after 3 h-incubation (Figure 1A). In HT-29 cells, the highest level of the total apoptotic population was observed after 6 h-treatment with an extract from 5-ISA-fortified lettuce and amounted to about 58%. Importantly, 5-ISA-fortified lettuce was the most potent as compared with other lettuce extracts. Moreover, in HT-29 colon cancer cells, we observed that exposure to synthetic forms of iodosalicylates, resulted in a significant increase in total apoptotic cells, relative to negative control and control lettuce (Figure 1B). This effect was not observed in AGS cells, in which treatment with synthetic 5-ISA and 3,5-diISA as well as lettuce biofortified with inorganic KIO_3_ led to a similar increase in total apoptotic cells as compared to control lettuce (Figure 1A).

### 2.2. *Lettuce Extracts Reduce Mitochondrial Membrane Potential*

**Figure 2:**
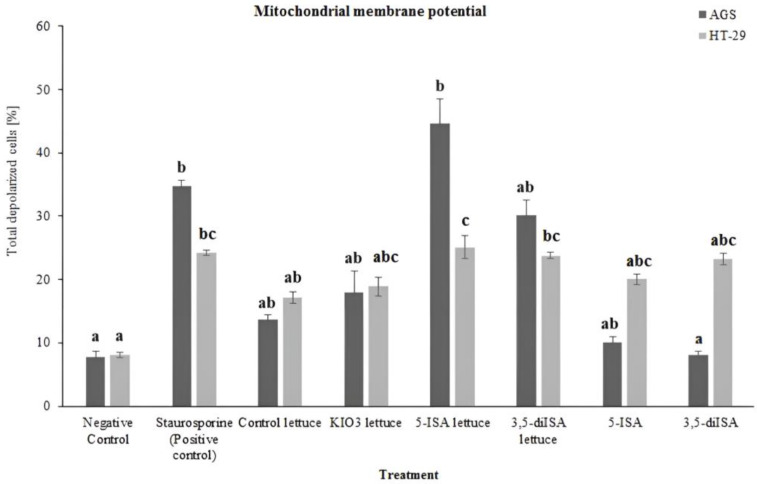
The effect of extracts from iodine-biofortified lettuce on mitochondrial membrane potential in human gastrointestinal cell line AGS and colorectal adenocarcinoma cell line HT-29. Cells were treated for 24 h with 1000 µg/mL extracts from control lettuce, or KIO_3_-fortified lettuce, or 5-ISA-fortified lettuce, or 3,5-diISA-fortified lettuce, or synthetic 5-ISA, or synthetic 3,5-diISA, or 1.5 µM staurosporine as positive control. Statistical significance was assessed using the Kruskal–Wallis non-parametric test followed by the multiple comparison post hoc test. Columns with the same letter are not significantly different (*p* > 0.05). Different letters indicate statistically significant differences at *p* < 0.05.

Since the assessment of apoptosis showed that extracts from iodine-biofortified lettuce lead to the induction of programmed cell death in gastric and colon cancer cells, we measured mitochondrial membrane potential to assess whether the observed apoptosis is linked to the mitochondrial pathway. Using the Muse™ MitoPotential Assay (Merck Millipore, Billerica, MA, USA), the mitochondrial membrane potential was measured in AGS and HT-29 cells exposed to 1000 µg/mL extracts from lettuce biofortified with inorganic and organic forms of iodine as well as to synthetic iodosalicylic acids. The representative dot plots were shown in Supplementary Figure S2. In AGS cells, 24 h exposure to 5-ISA- and 3,5-diISA-fortified lettuce resulted in 47% and 32% of the cell population with depolarized organelles, as compared to 15% of depolarized cells in non-enriched control lettuce (Figure 2). However, a statistically significant increase in total depolarized cells was observed only after exposure to 5-ISA-enriched lettuce when compared with negative control. Interestingly, a similar effect was detected in HT-29 colon cancer cells, where only treatment with extracts of lettuce enriched with 5-ISA caused a statistically significant increase from 10% to 27% in the number of cells exhibiting a depolarized mitochondria membrane, as compared with negative control. Additionally, we found that AGS gastric cancer cells were characterized by higher levels of total depolarized cells when compared with HT-29 colon cancer cells upon exposure to 5-ISA- and 3,5-diISA-fortified lettuce.

### 2.3. Lettuce Extracts Impact Expression of Apoptosis-Associated Proteins

**Figure 3:**
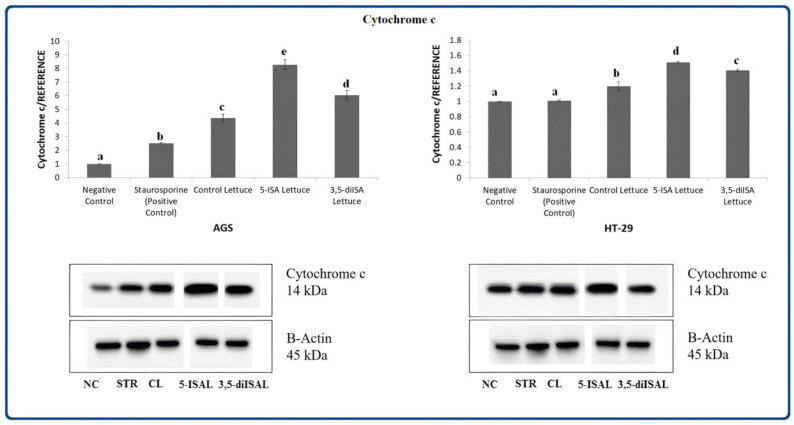
The effect of extracts from iodine-biofortified lettuce on cytochrome c expression in human gastrointestinal cell line AGS and colorectal adenocarcinoma cell line HT-29. Cells were treated for 48 h with 1000 µg/mL extracts from control lettuce (CL), or 5-ISA-fortified lettuce (5-ISAL), or 3,5-diISA-fortified lettuce (3,5-diISAL), or 1.5 µM staurosporine (STR) as a positive control. Untreated cells were used as negative control (NC). Densitometric analysis of obtained results was performed using ImageJ software Version 1.53t 24 August 2022 (National Institutes of Health, Bethesda, MD, USA). The results are shown as the mean ± SD normalized to the internal reference protein (B-actin). Statistical significance was assessed using one-way ANOVA followed by Tukey’s post hoc. Columns with the same letter are not significantly different (*p* > 0.05). Different letters indicate statistically significant differences at *p* < 0.05.

**Figure 4:**
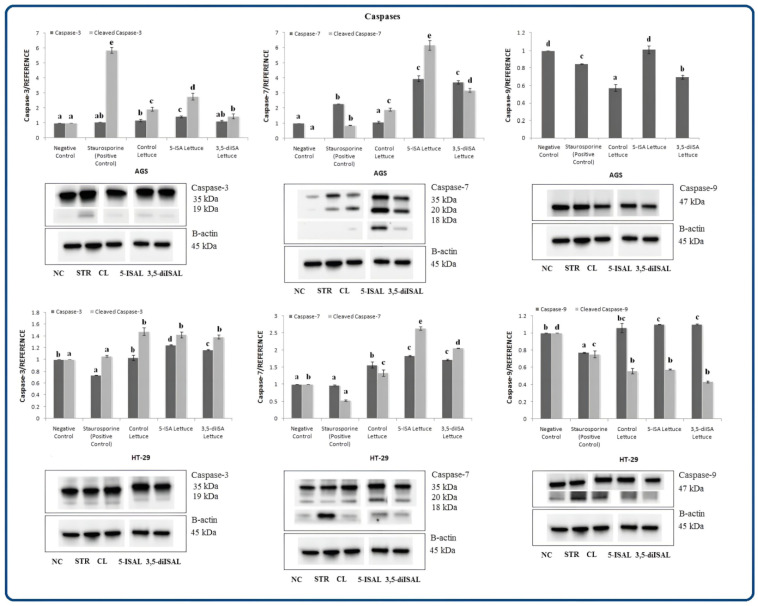
The effect of extracts from iodine-biofortified lettuce on caspase protein expression in human gastrointestinal cell line AGS and colorectal adenocarcinoma cell line HT-29. Cells were treated for 48 h with 1000 µg/mL extracts from control lettuce (CL), or 5-ISA-fortified lettuce (5-ISAL), or 3,5-diISA-fortified lettuce (3,5-diISAL), or 1.5 µM staurosporine (STR) as a positive control. Untreated cells were used as negative control (NC). Densitometric analysis of obtained results was performed using ImageJ software Version 1.53t 24 August 2022 (National Institutes of Health, Bethesda, MD, USA). The results are shown as the mean ± SD normalized to the internal reference protein (B-actin). Statistical significance was assessed using one-way ANOVA followed by Tukey’s post hoc. Columns with the same letter are not significantly different (*p* > 0.05). Different letters indicate statistically significant differences at *p* < 0.05.

**Figure 5:**
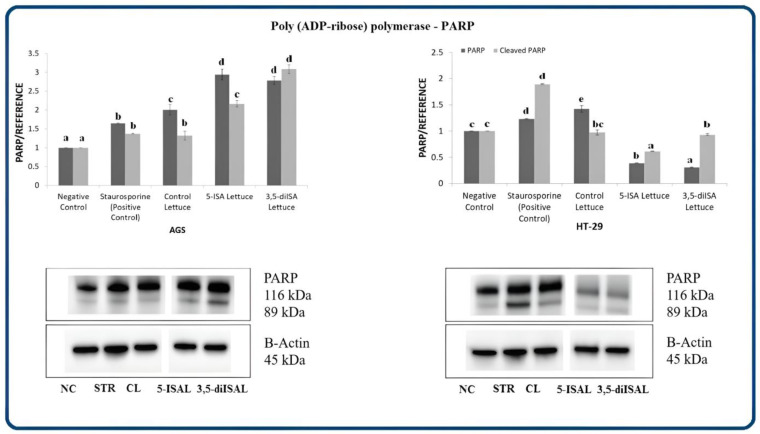
The effect of extracts from iodine-biofortified lettuce on poly (ADP-ribose) polymerase PARP expression in human gastrointestinal cell line AGS and colorectal adenocarcinoma cell line HT-29. Cells were treated for 48 h with 1000 µg/mL extracts from control lettuce (CL), or 5-ISA-fortified lettuce (5-ISAL), or 3,5-diISA-fortified lettuce (3,5-diISAL), or 1.5 µM staurosporine (STR) as a positive control. Untreated cells were used as negative control (NC). Densitometric analysis of obtained results was performed using ImageJ software Version 1.53t 24 August 2022 (National Institutes of Health, Bethesda, MD, USA). The results are shown as the mean ± SD normalized to the internal reference protein (B-actin). Statistical significance was assessed using one-way ANOVA followed by Tukey’s post hoc. Columns with the same letter are not significantly different (*p* > 0.05). Different letters indicate statistically significant differences at *p* < 0.05.

**Figure 6:**
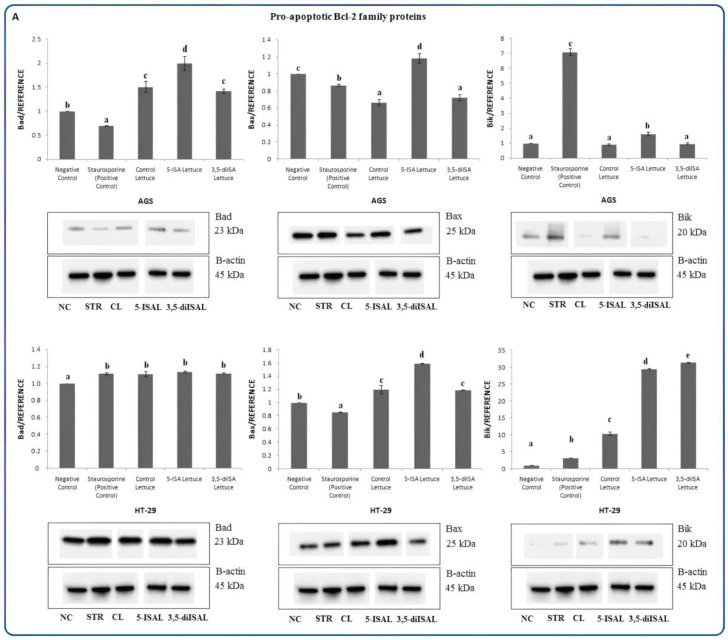

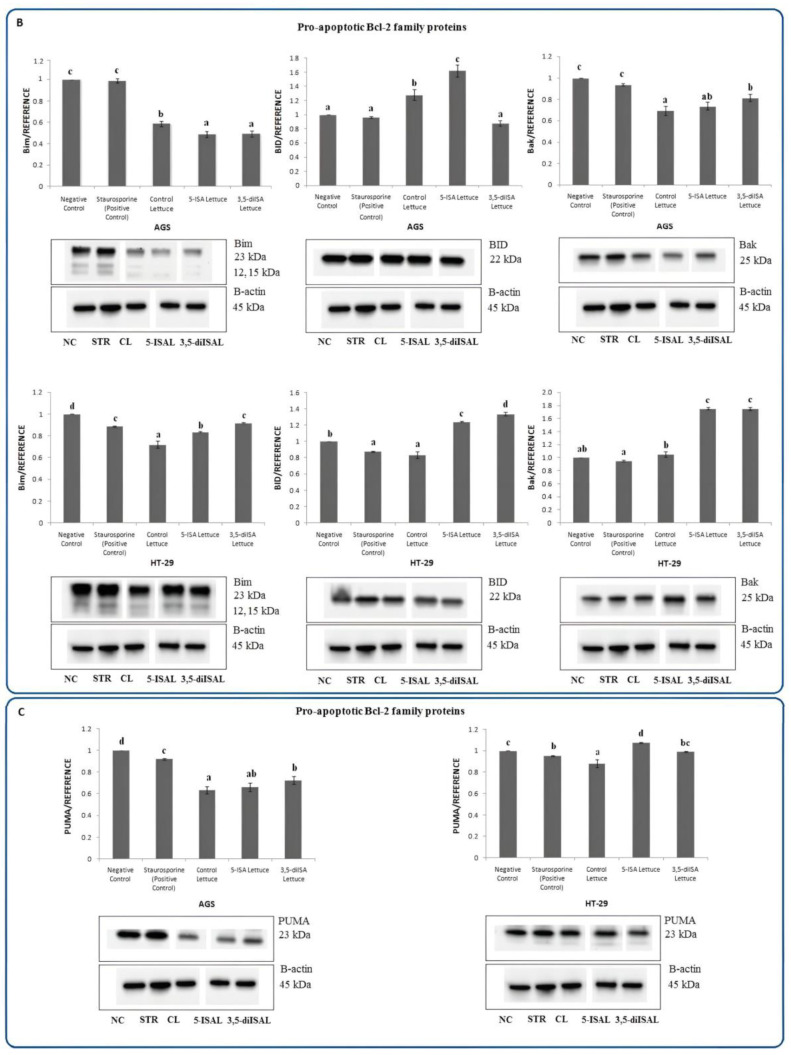
The effect of extracts from iodine-biofortified lettuce on pro-apoptotic Bcl-2 family proteins expression (**A**–**C**) in human gastrointestinal cell line AGS and colorectal adenocarcinoma cell line HT-29. Cells were treated for 48 h with 1000 µg/mL extracts from control lettuce (CL), or 5-ISA-fortified lettuce (5-ISAL), or 3,5-diISA-fortified lettuce (3,5-diISAL), or 1.5 µM staurosporine (STR) as a positive control. Untreated cells were used as negative control (NC). Densitometric analysis of obtained results was performed using ImageJ software Version 1.53t 24 August 2022 (National Institutes of Health, Bethesda, MD, USA). The results are shown as the mean ± SD normalized to the internal reference protein (B-actin). Statistical significance was assessed using one-way ANOVA followed by Tukey’s post hoc. Columns with the same letter are not significantly different (*p* > 0.05). Different letters indicate statistically significant differences at *p* < 0.05.

**Figure 7:**
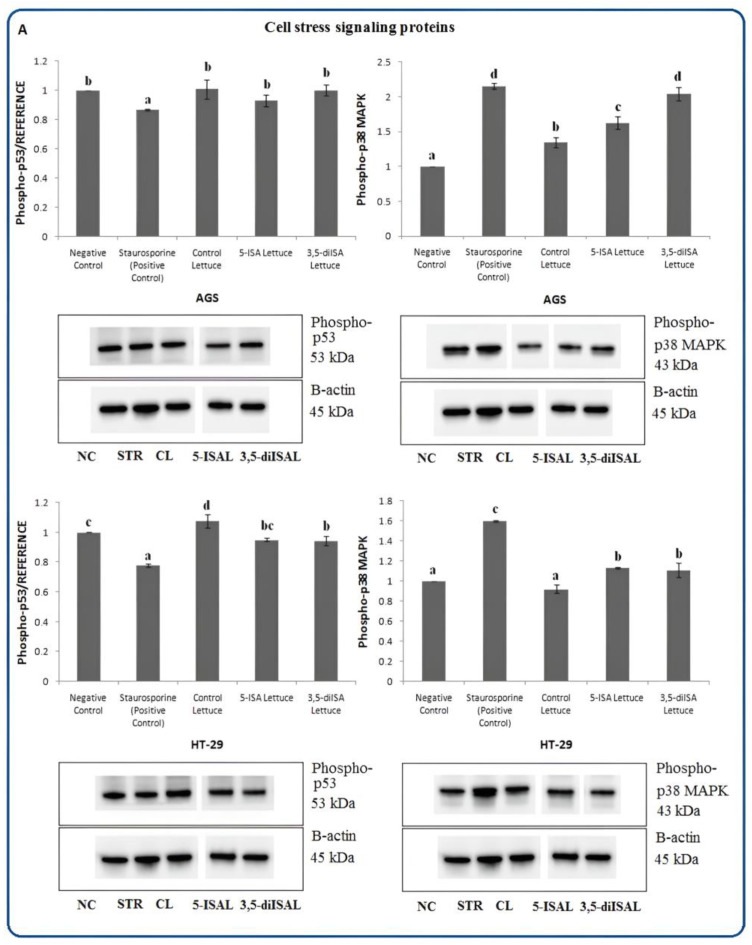

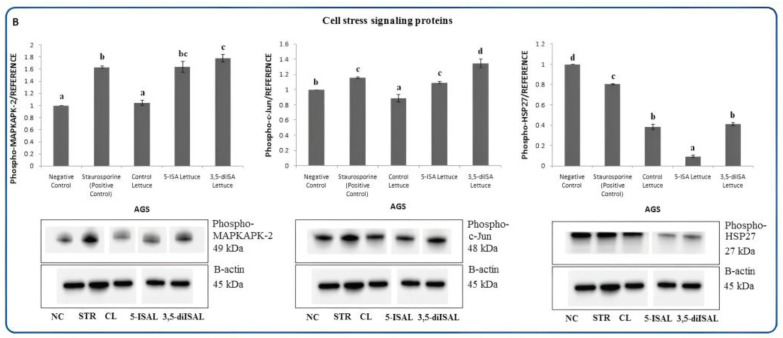
The effect of extracts from iodine-biofortified lettuce on cell stress signaling proteins expression (**A**,**B**) in human gastrointestinal cell line AGS and colorectal adenocarcinoma cell line HT-29. Cells were treated for 48 h with 1000 µg/mL extracts from control lettuce (CL), or 5-ISA-fortified lettuce (5-ISAL), or 3,5-diISA-fortified lettuce (3,5-diISAL), or 1.5 µM staurosporine (STR) as a positive control. Untreated cells were used as negative control (NC). Densitometric analysis of obtained results was performed using ImageJ software Version 1.53t 24 August 2022 (National Institutes of Health, Bethesda, MD, USA). The results are shown as the mean ± SD normalized to the internal reference protein (B-actin). Statistical significance was assessed using one-way ANOVA followed by Tukey’s post hoc. Columns with the same letter are not significantly different (*p* > 0.05). Different letters indicate statistically significant differences at *p* < 0.05.

**Figure 8:**
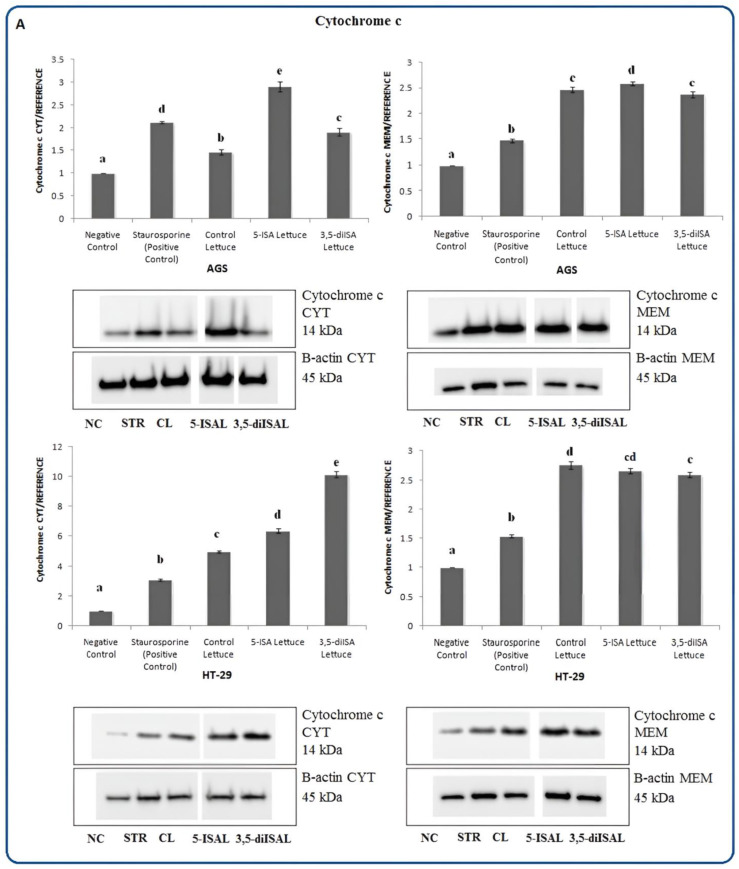

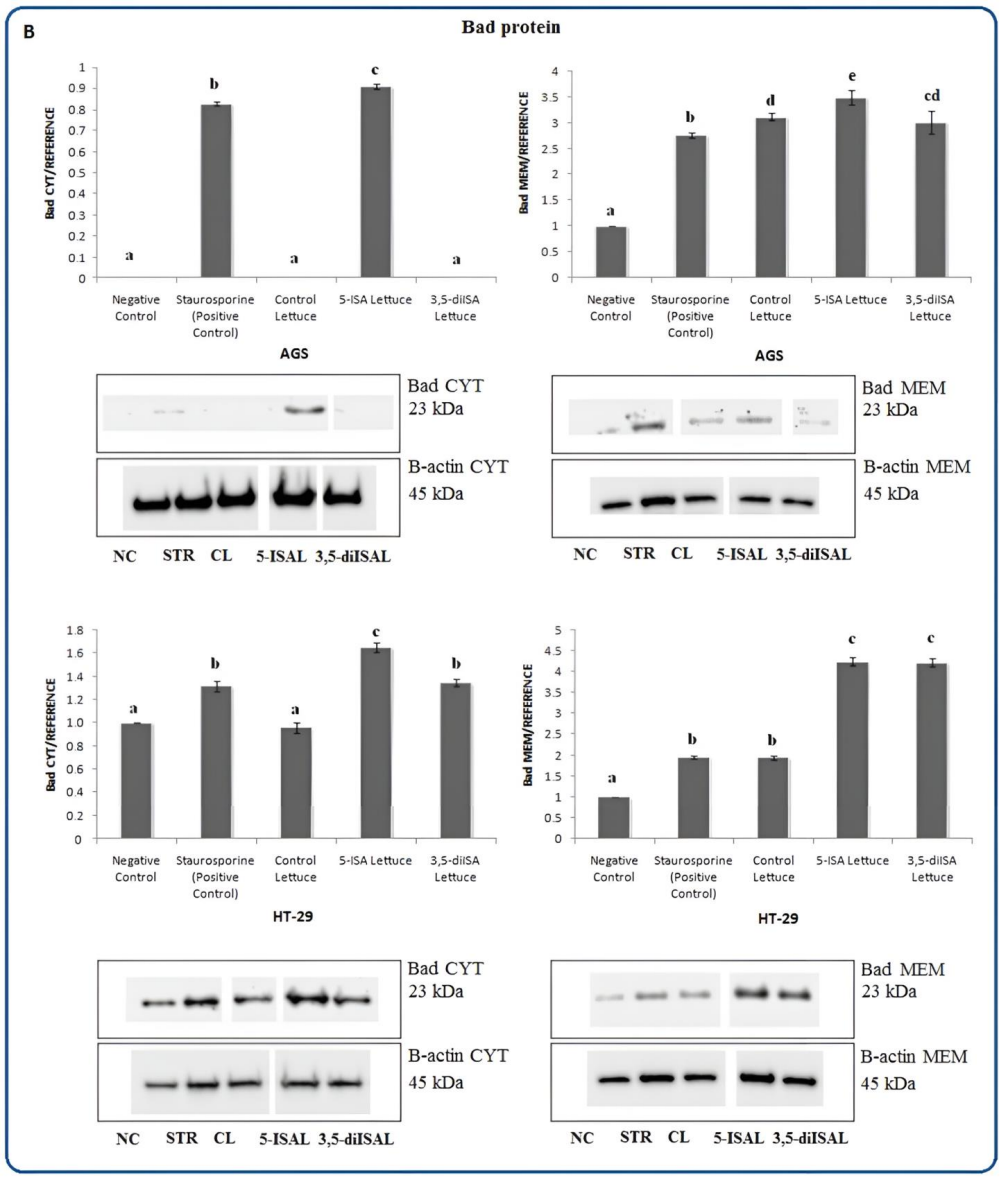
The effect of extracts from iodine-biofortified lettuce on cytochrome c (**A**) and Bad (**B**) proteins expression in the membrane (MEM) and cytoplasmic (CYT) fractions of human gastrointestinal cell line AGS and colorectal adenocarcinoma cell line HT-29. Cells were treated for 48 h with 1000 µg/mL extracts from control lettuce (CL), or 5-ISA-fortified lettuce (5-ISAL), or 3,5-diISA-fortified lettuce (3,5-diISAL), or 1.5 µM staurosporine (STR) as a positive control. Untreated cells were used as negative control (NC). Densitometric analysis of obtained results was performed using ImageJ software Version 1.53t 24 August 2022 (National Institutes of Health, Bethesda, MD, USA). The results are shown as the mean ± SD normalized to the internal reference protein (B-actin). Statistical significance was assessed using one-way ANOVA followed by Tukey’s post hoc. Columns with the same letter are not significantly different (*p* > 0.05). Different letters indicate statistically significant differences at *p* < 0.05.

Following the assessment of apoptosis as well as mitochondrial membrane potential, the expression analyses of stress and apoptosis signaling key proteins were performed to further assess molecular mechanisms of cancer cell death. Using Western blot as shown in Figure 3, Figure 4, Figure 5, Figure 6, Figure 7 and Figure 8, we assessed changes in the expression level of multiple proteins in AGS and HT-29 cells after 48 h exposure to extracts from iodine-enriched lettuce. Since we observed more profound effects of lettuce biofortified with iodosalicylates on the regulation of biological processes such as apoptosis and reduction of mitochondrial membrane potential, measurements of protein expression were performed in cells treated with extracts from 5-ISA- and 3,5-diISA-fortified lettuce. The extension of incubation time to 48 h during protein expression analysis was caused by a relatively small increase in the activation of caspases after iodine-fortified lettuce extracts, especially in the HT-29 cell line, as we showed in our previous research [19]. Extracts from lettuce fortified with 5-ISA substantially increased the level of cytochrome c which is released from the mitochondria and triggers the activation of caspase-dependent mitochondrial apoptotic pathway. Cytochrome c was upregulated by 728% and 51% upon 5-ISA-fortified lettuce in AGS and HT-29 cells, respectively, as compared to the negative control (Figure 3). Upregulation of this mitochondrial protein was also observed in AGS and HT-29 cells after treatment with 3,5-diISA and non-fortified lettuce (control lettuce) but to a lesser extent.

Figure 4 shows protein levels of caspases, including initiator (caspase-9) and effector (caspase-3 and -7) proteins, which are involved in programmed cell death. Protein level analysis confirmed a statistically significant 1.83-fold and 1.5-fold upregulation of caspase-7 after treatment of HT-29 cells with lettuce enriched with organic iodine and control lettuce, respectively, as compared with negative control. An increase in caspase-3 and caspase-9 was observed for 5-ISA- and 3,5-diISA-fortified lettuce but not control lettuce in this cell line (Figure 4). On the other hand, in AGS cells, only caspase-7 upon 5-ISA- and 3,5-diISA-fortified lettuce and caspase-3 upon control lettuce and 5-ISA-fortified lettuce showed a high and statistically significant increase in protein expression as compared to the negative control. Caspase-9 was upregulated in response to both 5-ISA- and 3,5-diISA-fortified lettuce but only vs. control lettuce but not vs. negative control (Figure 4). Importantly, our results confirmed the presence of a cleaved form of effectors caspases such as caspase-3 and -7 in both tested cell lines, suggesting induction of apoptosis (Figure 4, Western blots).

We also detected differential effects of the extracts on the activation of nuclear enzyme poly (ADP-ribose) polymerase PARP upon 48 h exposure (Figure 5). In AGS cells, PARP protein expression level was significantly increased by 1.5–3-fold vs. negative control upon each of the tested extracts, whereas only control lettuce led to a 42% PARP increase in HT-29 cells. Importantly, cleavage of PARP, a hallmark of apoptosis, was observed upon treatment with extracts from iodine-fortified lettuce in AGS cells, suggesting that fortified lettuce extracts induce apoptosis through the inactivation of PARP protein (cleavage) (Figure 5, Western blots). A cleaved form was also detectable upon control lettuce in HT-29 cells. Strikingly, PARP levels were highly decreased upon 5-ISA- and 3,5-diISA-fortified lettuce in HT-29 cells.

The Bcl-2 family contains proteins that are involved in the regulation of programmed cell death by controlling the mitochondrial membrane permeability and releasing pro-apoptotic factors, including cytochrome c. In AGS cells, 48 h exposure to the extract from 5-ISA-enriched lettuce led to a statistically significant 1.18–2-fold upregulation of pro-apoptotic proteins such as Bad, Bax, and BID, as compared to negative control and control lettuce (Figure 6A,B). However, upon 3,5-diISA-fortified lettuce, only Bad was overexpressed and this was observed only when compared to negative control but not versus control lettuce. Contrary to AGS cells, the extracts from 5-ISA- and 3,5-diISA-fortified lettuce upregulated Bik and Bak expression in the HT-29 cell line, as compared to negative control and control lettuce. Of note, both Bik and Bak were downregulated in AGS cells upon lettuce fortified with the organic forms of iodine. This finding could suggest that distinct pro-apoptotic proteins participate in apoptosis induction depending on the cancer type. In addition, in HT-29 cells, 5-ISA- and 3,5-diISA-fortified lettuce led to upregulation of Bax and BID as compared to the negative control (both extracts) or control lettuce (except for 3,5-diISA form) (Figure 6A,B).

Furthermore, to investigate whether cell death is triggered via p53-dependent or -independent mechanisms, we assessed the protein level of PUMA, which is a p53-upregulated modulator of apoptosis (Figure 6C), and the phosphorylation level of p53 tumor suppressor in AGS and HT-29 cells (Figure 7A). The increase in PUMA protein level was detected only in HT-29 cells after 48 h-incubation with extracts from 5-ISA-enriched lettuce, whereas no change was noted for 3,5-diISA-biofortified lettuce and a slight decrease upon control lettuce, as compared to the negative control. In AGS cells, all three extracts led to the downregulation of PUMA by approximately 34% as compared to the negative control. Thus, these findings confirm our above observation that distinct pro-apoptotic proteins are implicated in apoptotic pathways in different cancer cells and suggest the differences in apoptotic pathways between AGS and HT-29 cell lines. Interestingly, no changes in the level of phosphorylated p53 were revealed after treatments with either 5-ISA- or 3,5-diISA-biofortified lettuce extracts in the two tested cell lines, as compared to non-treated cells (negative control) (Figure 7A). Altogether, it indicates p53-independent apoptosis. Importantly, PUMA, a pro-apoptotic member of the Bcl-2 family, which was upregulated upon 5-ISA-enriched lettuce in HT-29 cells, has been reported to induce apoptosis in both p53-dependent and -independent fashion in cancer cells [20].

The p38 mitogen-activated protein kinase (p38 MAPK) pathway is responsible for many cellular processes such as apoptosis or resistance to apoptosis, cell differentiation, cell division, and transducing an environmental stress signal to the cells. It may act as a tumor suppressor by inhibiting cancer cell proliferation or as an oncogene by promoting cell cycle progression [21,22]. We have observed a statistically significant increase in phosphorylated p38 MAPK in AGS and HT-29 cells upon lettuce fortified with organic iodine. In AGS gastric cancer cells, phospho-p38 MAPK was upregulated by approximately 63% and 104% upon 5-ISA- and 3,5-diISA-fortified lettuce, respectively, compared with negative control (Figure 7A). Treatment of HT-29 cells with these extracts resulted in an increase in p38 MAPK expression by 13% and 12% vs. negative control (Figure 7A). To verify the signaling pathway involved in the regulation of apoptosis, the activation of substrates of p38 MAPK was evaluated, including MAP kinase-activated protein kinase 2 (MAPKAPK-2) and c-Jun, which are phosphorylated and activated by p38 MAPK. Both phospho-MAPKAPK-2 and phospho-c-Jun were detected only in AGS cells. The extracts from lettuce biofortified with organic forms of iodine led to 1.1–1.79-fold upregulation of phospho-MAPKAPK-2 and phospho-c-Jun, as compared to untreated cells (negative control) (Figure 7B). Similarly, the HSP27 heat shock protein, which protects cells against programmed death, was detected only in gastric cancer cells, where 60–90% downregulation of phospho-HSP27 was observed after treatments with 5-ISA and 3,5-diISA-biofortified lettuce extracts, as compared with negative control.

To determine the localization of proteins involved in apoptosis and test a hypothesis that the iodine-fortified lettuce induces the movement of these proteins, we performed a Western blot analysis using cytoplasmic and membrane cell fractions. The analysis revealed that in untreated AGS and HT-29 cells, cytochrome c was mainly accumulated in the membrane fraction and this fraction increased to a similar extent after treatments with all extracts from lettuce (Figure 8A). It is, however, important to note that higher levels of cytochrome c were also detected in the cytosolic fraction upon 5-ISA and 3,5-diISA-fortified lettuce, as compared to control lettuce and untreated cells. Cytochrome c release may be promoted by Bad, a pro-apoptotic member of the Bcl-2 protein family, which translocates to the mitochondrial outer membrane during apoptosis. Indeed, we showed that treatments with extracts from lettuce fortified with organic forms of iodine resulted in a substantial accumulation of Bad protein in the membrane fraction in AGS as well as in HT-29 cells (Figure 8B).

## 3. Discussion

Cancer prevention should be an equally important aspect of healthcare as therapeutic actions, since it is estimated that between 30 and 50% of all cancer cases are preventable, according to data from the World Health Organization. Despite this fact, preventive measures against cancer are consistently underutilized and underdeveloped [23]. One of the major modifiable risk factors for cancer, including gastric and colon cancer, is diet. Research on the health implications of dietary bioactive compounds clearly points out that healthy dietary patterns play a key role in relation to chronic disease risk reduction. There is strong scientific evidence of the beneficial effects of plant-based dietary patterns that are low in red and processed meat towards the reduction of colorectal cancer risk [24]. As found in vitro and in vivo studies, dietary phytochemicals, such as polyphenols, exert anticancer effects through inhibition of angiogenesis, proliferation, and cell cycle progression in the colon [23,24,25]. Moreover, multiple diet-derived phytochemicals can modulate the intestinal microbiota that plays an important and direct role in the promotion of healthy colon epithelium [26].

Our previous findings showed that extracts from lettuce fortified with iodine, that largely contain an enhanced content of polyphenolic compounds, have the ability to reduce colon and gastric cancer cell viability and proliferation. Therefore, in this study, we have focused on investigating potential molecular mechanisms responsible for the inhibition of AGS and HT-29 cell growth upon treatments with iodine-enriched lettuce extracts. Pro-apoptotic effect of molecular iodine (I_2_) was studied in different cancer cell lines; however, to our best knowledge, it is the first study to explain the pro-apoptotic effect of lettuce fortified with the organic form of iodine: 5-ISA and 3,5-diISA.

The induction of programmed cell death through plant-derived compounds is a promising strategy for anticancer therapy [27]. The obtained results point to a high pro-apoptotic potential of iodine-fortified lettuce in gastric and colon cancer cells. Our results revealed the capacity of extracts from iodine-fortified lettuce to induce apoptosis and promote depolarization of the mitochondrial membrane in both tested cell lines, AGS and HT-29. Shrivastava et al. [13] also demonstrated that molecular iodine (I_2_) induces apoptosis in numerous human breast cancer cell lines, and the iodine treatment resulted in the dissipation of the mitochondrial membrane potential in cancer cells. Of note, our study showed a statistically significant rise in the number of depolarized cells only upon 5-ISA-enriched lettuce which may indicate stronger signaling that stimulates the mitochondrial apoptosis pathway (Figure 2).

Our previous results suggested that the treatment of cancer cells with iodine-fortified lettuce induces apoptosis via mitochondrial pathways. We showed changes in the expression pattern of genes involved in cell death and a reduction in the activity of the anti-apoptotic Bcl-2 protein [19].

To confirm our earlier assumptions of the involvement of mitochondria in cell death upon the extracts, we here measured the expression of proteins involved in apoptosis. In our experiment, Western blot analyses indicated that extracts from iodine-enriched lettuce increased the expression of cytochrome c in AGS and HT-29 cell lines (Figure 3). The levels of cytochrome c were around 15-fold higher upon treatments in AGS cells than in HT-29 cells. Moreover, we also detected the presence of cytochrome c in a cytosol compartment which suggested the possible effects of studied extracts on the outer mitochondrial permeabilization (Figure 8A).

The literature describes two different potential mechanisms of iodine-induced apoptosis in cancer cell lines. A direct action, which is based on the dissipation of the mitochondrial membrane potential through oxidized iodine, and an indirect effect, where cell death is induced by the generation of iodolipids or the activation of peroxisome proliferator-activated receptors type gamma (PPARγ) [28]. In vitro and in vivo studies indicated that supplementation with iodine has antitumor activities in human breast cancer [13,14]. It was found that the antiproliferative effects of molecular iodine in MCF-7 breast cancer cells may be mediated by iodine-containing lipids [29]. Nuñez-Anita et al. [29] confirmed that iodine treatment generates iodolipids that reduce breast cancer cell proliferation through the induction of programmed cell death. As demonstrated in studies on rats, I_2_ caused an increase in 6-iodolactone (6-IL) formation in tumoral mammary tissue [14]. Furthermore, the concentrations of arachidonic acid (AA), which is a substrate for 6-IL formation, were also much higher in breast cancer cells as compared to normal cells [14,29]. 6-IL acts as a specific ligand of PPARγ that plays an important role in the reduction of cell proliferation and may induce cell death in many types of cancer [30]. Importantly, it has been reported that the activation of PPARγ results in a reduction of survivin and may induce apoptosis via caspase-3 [31]. Nava-Villalba et al. [32] showed that antiproliferative and pro-apoptotic effects of 6-IL result from its ability to inhibit migration of MCF-7 cells, which is dependent on PPARγ activation.

Previous studies provide evidence suggesting that various signaling pathways are activated during iodine-induced apoptosis. Arroyo-Helguera et al. [15] demonstrated that triggering programmed cell death by iodine in MCF-7 breast cancer cells is mediated by the BAX-caspase and/or AIF/PARP1 pathways. On the other hand, Shrivastava et al. [13] suggested that the pro-apoptotic effects of molecular iodine in breast cancer are caspase pathway-independent. In our present study, we observed the increased expression of initiator caspase-9 in response to extracts from iodine-fortified lettuce vs. control lettuce in AGS cells and vs. negative control in HT-39 cells (Figure 4). Caspases are believed to be crucial for the execution pathway during apoptosis [33]. Activated caspase-9 is responsible for the proteolytical cleavage of downstream effector caspases such as caspase-3 and -7, resulting in their activation [34]. Indeed, we found that extracts from iodine-fortified lettuce increase levels of caspase-3 and -7 (Figure 4), which are classified as executioners [35]. Interestingly, western blots depict an obvious rise in the activated cleaved form of caspase-7 in both cell lines (Figure 4). Hence, our results indicate the involvement of the caspase pathway in iodine-induced apoptosis in colon and gastric cancer cells. Similar findings were obtained by Soriano et al. [36], who demonstrated that the supplementation with molecular iodine or potassium iodide increases PPARγ and caspase-3 expression and thus triggers caspase-mediated apoptosis pathways in mammary cancer. Other authors reported the effects of iodine on the proliferation and apoptosis of human papillary-thyroid carcinoma cells [37]. In addition, they showed that expression levels of caspase-3 in cancer tissue are positively correlated with urinary iodine concentrations and the correlation is dose-dependent.

Poly (ADP-Ribose) Polymerase (PARP), which is responsible for maintaining DNA stability and DNA repair, is also one of the key substrates cleaved by effector caspases [35]. PARP was originally considered as caspase-3 substrate; however, it is now known, that caspase-7 is also involved in the cleavage of PARP [38,39]. Once cleaved, PARP loses its activity which brings about the inhibition of DNA repair and consequently results in apoptosis. In our present work, the treatment with iodine-fortified lettuce led to an increase in PARP expression in the AGS cell line. Interestingly, this effect was not observed in HT-29 cancer cells, where the treatments with lettuce extracts significantly decreased the level of PARP (Figure 5). However, it is important to note that in response to lettuce extracts in AGS cells, we detected the presence of an 89-kDA PARP-cleaved fragment, that can be considered as an apoptosis marker (Figure 5). Our findings indicate the functional activation of effector caspases in both cell lines and are consistent with previous research, showing iodine-induced proteolytic cleavage of PARP1 in MCF-7 cancer cells [15].

Apart from iodine, the fortified lettuce extracts contain polyphenols, highly bioactive compounds. Our previous studies showed that iodine-fortified lettuce contains elevated concentrations of chlorogenic acid, sinapic acid, *p*-coumaric acid, ferulic acid, hippuric acid, protocatechuic acid, and 3-hydroxybenzoic acid, in comparison to control lettuce [18]. The association between dietary polyphenol consumption and the risk of developing cancer has been the subject of numerous studies in various experimental models and promising effects have been reported in gastrointestinal cancer and other tumor types such as breast, prostate, ovarian, or lung cancer [40]. Therefore, we suggest that the pro-apoptotic effects of iodine-fortified lettuce could be enhanced by the high level of polyphenolic compounds. For instance, the induction of apoptosis via Bax upregulation and Bcl-2 downregulation was found in MCF-7 breast cancer cells treated with quercetin [41]. Other authors demonstrated that kaempferol triggers pro-apoptotic effects by inducing cell cycle arrest in the G2/M phase in ovarian cancer cells [42]. In our previous study, we indeed observed that iodine-fortified lettuce extracts led to the reduction of the activity of anti-apoptotic Bcl-2 protein and caused cell cycle arrest in the G2/M phase [19]. Dysregulation of the Bcl-2 family of proteins, which is often observed in a wide variety of tumors, may facilitate tumor development and resistance to cancer therapy [41,42,43]. Therefore, it is essential to study the effects on the balance between pro- and anti-apoptotic members of the Bcl-2 family protein [43]. Since our previous study addressed the activity of anti-apoptotic Bcl-2 [19], herein we analyzed the effect of fortified lettuce extracts on pro-apoptotic proteins from the Bcl-2 family and apoptotic pathways.

In the present work, we observed that pro-apoptotic proteins, Bad, Bax, and BID, are highly expressed in AGS and HT-29 cancer cells in response to 5-ISA- and 3,5-diISA-fortified lettuce vs. negative control (except BID after 3,5-diISA in AGS) (Figure 6A,B). Our findings are consistent with reports by other authors, who demonstrated increased Bax expression accompanied by endogenous Bcl-2 degradation in iodine-treated human breast cancer cells [13]. Interestingly, our results revealed that iodine-induced cell death is independent of the levels of Bik and Bak in AGS cells, whereas both Bik and Bak were profoundly upregulated in HT-29 cells upon fortified lettuce (Figure 6A,B). Our analysis shows that distinct members of the Bcl-2 family may be induced to execute an iodine-triggered cell death program depending on the type of cancer. Bad and BID, which together with Bik, Bim, and Puma belong to the pro-apoptotic BH3-only Bcl-2 family of proteins that are believed to be essential initiators of apoptosis and responsible for the promotion of the mitochondrial outer membrane permeabilization through the activation the effector proteins, Bax and Bak [44]. Bad as opposed to other Bcl-2 family members does not have a C-terminal transmembrane domain which enables anchoring to the outer mitochondrial membrane and nuclear envelope [45]. Depending on the phosphorylation state, Bad can function as a pro-apoptotic or pro-survival factor. It was found that phosphorylation of Bad may promote drug resistance and survival of cancer cells whereas lack of phosphorylation modification on Bad results in a subcellular relocation of Bad to the mitochondria and triggering apoptosis by releasing cytochrome c to the cytosol [44,45,46]. Our results suggest that the mitochondria-mediated intrinsic apoptosis is induced by extracts from iodine-fortified lettuce via the activation and translocation of the non-phosphorylated form of Bad protein into the membrane fraction (Figure 8B).

We have previously reported increased ROS production in response to iodine-fortified lettuce extracts and synthetic forms of iodosalicylic acid in HT-29 and AGS cells [18]. Other studies have shown that elevated oxidative intracellular stress leads to the activation of the p38 mitogen-activated protein kinase (MAPK) signaling pathway and consequently increased transcriptional activity of the p53 tumor suppressor gene [47,48]. The p38 mitogen-activated protein kinase (MAPK) is activated by dual phosphorylation of tyrosine and threonine residues in response to extracellular stimuli such as oxidative stress [49]. Activated p38 MAPK is responsible for the regulation of many different processes including apoptosis, cell division, cell invasion, and inflammatory response [50]. Hence, p38 MAPK signaling is believed to be an important indicator of the efficacy of anticancer therapeutics [51]. For instance, cisplatin, a commonly used anticancer drug, results in the activation of the p38 MAPK pathway in several human cancer cell lines [52]. Importantly, Liu et al. [53] reported that Asiatic acid (AA), extracted from *Centellaasiatica*, induces programmed cell death in cisplatin-resistant nasopharyngeal carcinoma cells through p38 phosphorylation and activation. In Caco2 colon cancer cells, p38 induced apoptosis through increasing pro-apoptotic Bim expression and inactivating pro-survival Bcl-2 members [54]. It is also known that activated p38 is involved in the cleavage of caspase-3 during programmed cell death and regulation of pro-apoptotic Bcl-2 proteins [55,56]. However, there are studies reporting the opposite role of p38 MAPK, where cancer cells were sensitized to cisplatin-induced apoptosis mediated by ROS and JNK upon inhibition of p38 MAPK [57]. Thus, the role of p38 MAPK signaling in cancer cells may be context-dependent (cell type or apoptosis inducers) [21].

Nevertheless, our present work provides evidence that iodine-fortified lettuce induces the mitochondrial pathway of apoptosis and this is accompanied by an increase in phospho-p38 MAPK levels. Treatment with 5-ISA- and 3,5-diISA-fortified lettuce extracts significantly increased the expression of phospho-p38 MAPK in colon and gastric cancer cells, as compared to control lettuce and non-treated cells (Figure 7A). In addition, in AGS gastric cancer cell line, we observed the activation of mitogen-activated protein kinase-activated protein kinase 2 (MAPKAPK-2) (Figure 7B), which is believed to be a downstream substrate and a direct target of p38 MAPK [58,59]. Simultaneously, expression of heat shock protein 27 (HSP27) was strongly reduced in gastric cancer cells in response to iodine-fortified lettuce extracts, with the strongest effect for 5-ISA-enriched lettuce, as compared to negative control and non-fortified lettuce (Figure 7B). HSP27 is reported to be a terminal substrate of the p38 MAPK cascade that may be involved in the inhibition of intrinsic as well as extrinsic apoptosis pathways [60,61]. It is known, that HSP27 contributes to cell survival through inhibition of key proteins, including cytochrome c-mediated activation of caspase-3, Bax, or BID, that are directly involved in cellular apoptosis [62,63]. Overexpression of HSP27, which is closely related to the progression of cancerous cells, resistance to treatments, and poor prognosis, was previously reported in gastric, colorectal, and liver cancer [64]. Zhu et al. [65] demonstrated that the activation of the p38/HSP27 pathway protects gastric cancer cells, GC-823 and MGC-803, from melatonin-induced suppression of cell proliferation.

Importantly, an increase in phospho-p38 levels was not accompanied by p53 activation upon treatments with iodine-fortified lettuce extracts (Figure 7A). Thus, our findings suggest that iodine-fortified lettuce-induced apoptosis is p53-independent. However, it is important to note that p53 is known to be involved in DNA repair, cell cycle regulation, and apoptosis. p53 may interact with members of the Bcl-2 family and activate many pro-apoptotic proteins, including PUMA [66,67]. PUMA, a p53-upregulated modulator of apoptosis, is believed to be one of the most efficient pro-apoptotic proteins that can trigger apoptosis and destroy cancer cells within a short period [68]. Importantly, our results showed a substantial increase in PUMA levels in HT-29 cells, a p53-mutant human colon cancer cell line, in response to 5-ISA-fortified lettuce (Figure 6C). Such effects were not observed in AGS gastric cancer cells. Although PUMA is a target of p53, it can also be induced by a p53-independent pathway [20], which could explain PUMA upregulation in HT-29 cells without changes in phospho-p53.

Our previous study revealed that 5-ISA-fortified lettuce leads to the activation of methylation-silenced tumor suppressor genes in HT-29 cells which could be linked to downregulation of MDM2 [19]. A proposed mechanism involves MDM2 downregulation upon 5-ISA-fortified lettuce which causes RB-dependent delocalization of DNMT3A methyltransferase from regulatory regions of tumor suppressor genes and thus DNA demethylation and transcriptional activation of those genes. Strikingly, MDM2 is functionally connected to PUMA that was upregulated by 5-ISA-fortified lettuce in our present study. Low MDM2 and high PUMA levels were found in cancer patients as favorable prognostic features [69]. Furthermore, the downregulation of MDM2 has been reported to enhance PUMA expression [70,71]. Hence, PUMA upregulation mediated by iodine-fortified lettuce could be the trigger for the epigenetic activation of tumor suppressor genes through the MDM2/RB/DNMT3A pathway.

In AGS gastric cancer cells, we also demonstrated the elevated level of phospho-c-Jun upon the lettuce extracts fortified with iodine (Figure 7B). Previous studies have shown that c-Jun, which is a component of the signal-transducing transcription factor of the activator protein-1 (AP-1) family, has been involved in cell survival as well as apoptosis, depending on the cell type and stimulus [72,73]. Even though c-Jun dysregulation and its oncogenic activity have been shown in many cancer types, there is also evidence summarizing that upregulation of c-Jun inhibits proliferation and induces cell death [74,75]. Studies have proven that c-Jun may mediate apoptosis by triggering caspase-mediated cleavage of proteins, which is consistent with our results [75,76]. In addition, p38 MAPK plays an important role in posttranslational c-Jun regulation through c-Jun phosphorylation at serines 63 and 73, consequently modulating the transcriptional activity of the AP-1 transcription factor complex [77]. Taylor et al. [72] suggested that the p38 MAPK signaling pathway and phosphorylation of c-Jun are involved in the induction of apoptosis in response to Ad-eIF5A1 infection in A549 lung cancer cells and this process may involve the AP-1 transcription factor complex. It was also shown that c-Jun is necessary for the induction of programmed cell death in response to cisplatin and cells derived from c-Jun knock-out mice are characterized by greater resistance to apoptosis as compared with wild-type cells [78].

In summary, our present study shows that extracts from lettuce fortified with organic forms of iodine lead to the induction of caspases and mitochondria-dependent pathways of apoptosis independently of p53, although distinct players are involved depending on the type of cancer and the form of iodine. Lettuce fortified with 5-ISA exerts the strongest effects, including those on depolarization of the mitochondrial membrane and PUMA upregulation which connects to previously reported epigenetic activity of the extract. Our findings strengthen the evidence for a broad spectrum of anticancer effects of extracts from iodine-fortified plants and determine several proteins, including PUMA, that could be targets of anticancer therapies.

## 4. Materials and Methods

### 4.1. Plant Material

The production of iodine-biofortified lettuce was performed in a greenhouse of the University of Agriculture in Krakow. The analyzed material was lettuce *L.sativa* cv. ‘Melodion’ fortified with inorganic, KIO_3_, and organic, 5-ISA and 3,5-diISA, forms of iodine, as previously described in detail [17,18]. Non-biofortified lettuce was used as control plants. The plants were grown in a hydroponic system (Nutrient Film Technique). The extraction process of biofortified plants was performed using 70% ethanol as an extraction solvent. Alcohol was removed by a rotary evaporator and next the extracts were lyophilized, frozen, and stored at −20 °C until used for further analyses.

### 4.2. Cell Culture

The human gastrointestinal cancer cell lines: gastric adenocarcinoma cell line AGS (ATCC^®^ CRL-1739™, Manassas, VA, USA) and human colorectal adenocarcinoma cell line HT-29 (ATCC^®^HTB-38™, Manassas, VA, USA) were obtained from the American Type Culture Collections (ATCC, Manassas, VA, USA). Cells were cultured under controlled conditions in an appropriate medium supplemented with 10% fetal bovine serum (FBS) according to the ATCC protocol.

### 4.3. Cell Treatment

To determine the effect of the lettuce extracts on mitochondrial membrane potential and apoptosis, AGS and HT-29 cells were seeded at a density of 3 × 10^5^ and 6 × 10^4^ cells per well in 6- and 24-well plates and cultured in a growth medium for 24 h. Subsequently, the growth medium was replaced with a medium containing synthetic iodosalicylic acids or extracts from control lettuce and lettuce biofortified with organic (5-ISA and 3,5-diISA lettuce) and inorganic (KIO_3_ lettuce) forms of iodine at the concentration of 1000 µg/mL. For the apoptosis analysis, cells were incubated with extracts and synthetic iodosalicylic acids for 1, 3, and 6 h, whilst for the mitochondrial potential membrane analysis cells were cultivated with tested extracts for 24 h.

To analyze protein expression, AGS, and HT-29 cells were seeded into dishes at 1 × 10^6^ cells per dish and cultured in a growth medium for 24 h. After this time, cells were treated with extracts from control and iodine-fortified lettuce for 48 h. For all experiments, non-treated cells were used as a negative control, whereas cells treated with staurosporine (1.5 µM for 3 h) served as a positive control to induce apoptosis.

### 4.4. Apoptosis Analysis

The Muse™ Annexin V & Dead Cell Kit (Merck Millipore, Billerica, MA, USA, Catalog No. MCH100105) was used for the quantitative analysis of live, early, and late apoptosis, and cell death. This assay utilizes Annexin V to detect phosphatidylserine (PS) on the external membrane of apoptotic cells. Application of 7-Aminoactinomycin D (7-AAD), which is a dead cell marker excluded from live as well as early apoptotic cells, allows to distinguish four populations of cells: non-apoptotic, early apoptotic, late stage apoptotic and dead cells, and mostly nuclear debris. The results of the assay were measured using the Muse™ Cell Analyzer (Merck Millipore, Billerica, MA, USA).

### 4.5. *Mitochondrial Membrane Potential Analysis*

To assess the effect of extracts from control and iodine-biofortified lettuce on the mitochondrial membrane potential, the Muse™ MitoPotential Assay (Merck Millipore, Billerica, MA, USA, Catalog No. MCH100110) was used. The assay utilizes a lipophilic dye and 7-Aminoactinomycin D (7-AAD) which allow for the determination of changes in the mitochondrial potential and cellular plasma membrane permeabilization. This assay provides information about the percentage of four populations of cells: live cells with depolarized mitochondrial membrane, live cells with intact mitochondrial membrane, dead cells with depolarized mitochondrial membrane, and dead cells with intact mitochondrial membrane. Analysis of mitochondrial membrane potential was performed using the Muse™ Cell Analyzer (Merck Millipore, Billerica, MA, USA).

### 4.6. Protein Expression Analysis

The cell lysates were obtained using Cell Lysis Buffer (#9803) and Cell Fractionation Kit (#9038) with the addition of Protease/Phosphatase Inhibitor Cocktail (#5872) according to the manufacturer’s instruction (Cell Signaling Technology, Danvers, MA, USA). The total protein was determined using Pierce^TM^ BCA Protein Assay Kit (Thermo Fisher Scientific, Waltham, MA, USA, Catalog No. 23227). The measurement was carried out using a plate reader Thermo Scientific™ Multiskan™ GO Microplate Spectrophotometer (Waltham, MA, USA). The protein extracts were loaded and separated on a 4–15% Mini-PROTEAN^®^ TGX Stain-Free™ Protein Gels (Bio-Rad Laboratories, Inc., Hercules, California, USA, #4568083). After the electrophoresis process, the Trans-Blot Turbo Transfer System with the Trans-Blot Turbo Mini 0.2 µm Nitrocellulose Transfer Packs (Bio-Rad Laboratories, Inc., Hercules, CA, USA, #1704158) was used to transfer the protein from a gel to a membrane. Next, membranes were blocked with 5% non-fat milk (in TBST buffer) to avoid non-specific binding of primary and secondary antibodies to the membrane. After blocking, the membranes were incubated with the appropriate primary antibody against: Bcl-2 associated agonist of cell death (Bad) (#9239), Bcl-2 antagonist/killer 1 (Bak) (#12105), Bcl-2 associated X, apoptosis regulator (Bax) (#5023), BH3 interacting-domain death agonist (BID) (#2002), Bcl-2-interacting killer (Bik) (#4592), Bcl-2-interacting mediator of cell death (Bim) (#2933), caspase-3 (#14220), cleaved caspase-3 (#9664), caspase-7 (#12827), cleaved caspase-7 (#8438), caspase-9 (#9508), cleaved caspase-9 (#52873), cytochrome c (#11940), p53 upregulated modulator of apoptosis (PUMA) (#12450), poly (ADP-ribose) polymerase (PARP) (#9542), cleaved PARP (#5625), phospho-HSP27 (#9709), phospho-c-Jun (#3270), phospho-MAPKAPK-2 (#3007), phospho-p53 (#9286), phospho-p38 MAPK (#4511) at 1:1000 dilution for 1 h at room temperature (Cell Signaling Technology, Danvers, MA, USA). Eventually, an appropriate anti-rabbit (#7074) or anti-mouse (#7076) secondary antibody conjugated to horseradish peroxidase was used (Cell Signaling Technology, Danvers, MA, USA). Detection of proteins was performed by chemiluminescence with Clarity™ Western ECL Substrate (Bio-Rad Laboratories, Inc., Hercules, CA, USA, #1705060). The ChemiDoc Imaging System (Bio-Rad Laboratories, Inc., Hercules, CA, USA) was applied to visualize the blotting images. Densitometric analysis of obtained results was performed using ImageJ software (National Institutes of Health, Bethesda, MD, USA). β-actin (Cell Signaling Technology, Danvers, MA, USA, #8457) was used as the internal reference protein for normalization. Due to the fact that samples were loaded in duplicate on the same gel, some strips were cut to show an exemplary blot.

### 4.7. Statistical Analysis

The statistical analysis was performed by using Statistica 13.1 PL program (StatSoft, Inc., Tulsa, OK, USA). The results of the analyses were expressed as mean ± standard deviation (SD) and all experiments were performed in at least three technical and biological replications. To evaluate the normality of distribution, the Shapiro-Wilk test was applied. Statistically significant differences in groups were compared using the one-way analysis of variance (ANOVA) followed by Tukey’s post hoc and the Kruskal–Wallis non-parametric test followed by the multiple comparison post hoc test.

## 5. Conclusions

The obtained results demonstrate that programmed cell death induced by lettuce fortified with organic forms of iodine may be triggered and executed through different signaling pathways, depending on the type of cells and the type of the organic iodine form. The findings show that mitochondrial apoptosis induced by iodine-fortified lettuce in gastric cancer cells may be carried out through the p38 MAPK/MAPKAPK-2/c-Jun signaling pathway where pro-apoptotic Bcl-2 family members, such as Bad, Bax, and BID, are involved followed by a mitochondrial release of cytochrome c. However, the programmed cell death in human colorectal cancer cells is mediated by another signaling pathway—p38 MAPK/PUMA without the involvement of MAPKAPK-2 and c-Jun. Our results support the hypothesis that organic forms of iodine such as 5-ISA and 3,5-diISA are important factors in the activation of mitochondrial apoptotic pathway in a p53-independent manner in AGS and HT-29 cancer cells.

## 6. Patents

The method of enriching plants with iodine with 5-iodosalicylic acid and 3,5-diiodosalicylic acid is patented by the Polish Patent Office—patent number P.410806 (20 November 2017).

## Data Availability

The authors will share the data upon request.

## Supplementary Materials

The following supporting information can be downloaded at: https://www.mdpi.com/article/10.3390/ijms24129869/s1.

## References

[1] Sung H., Ferlay J., Siegel R.L., Laversanne M., Soerjomataram I., Jemal A., Bray F. (2021). Global Cancer Statistics 2020: GLOBOCAN Estimates of Incidence and Mortality Worldwide for 36 Cancers in 185 Countries. CA Cancer J. Clin..

[2] Rajabi S., Maresca M., Yumashev A.V., Choopani R., Hajimehdipoor H. (2021). The Most Competent Plant-derived Natural Products for Targeting Apoptosis in Cancer Therapy. Biomolecules.

[3] Fernald K., Kurokawa M. (2013). Evading Apoptosis in Cancer. Trends Cell Biol..

[4] Fulda S. (2011). Targeting Apoptosis Signaling Pathways for Anticancer Therapy. Front. Oncol..

[5] Chen X., Pu X., Pu X., Li X., Liu Z., Mei M., Wang X., Zhang F., Qiu B., Yu J. (2022). Extracts of Knoxia Roxburghii (Spreng.) M. A. Rau Induce Apoptosis in Human MCF-7 Breast Cancer Cells via Mitochondrial Pathways. Molecules.

[6] Fouzat A., Hussein O.J., Gupta I., Al-Farsi H.F., Khalil A., Al Moustafa A.E. (2022). Elaeagnus Angustifolia Plant Extract Induces Apoptosis via P53 and Signal Transducer and Activator of Transcription 3 Signaling Pathways in Triple-Negative Breast Cancer Cells. Front. Nutr..

[7] Lem F.F., Cheong B.E., Teoh P.L. (2022). Ruellia Tuberosa Ethyl Acetate Leaf Extract Induces Apoptosis and Cell Cycle Arrest in Human Breast Cancer Cell Line, MCF-7. Sci. Pharm..

[8] D’Archivio M., Santangelo C., Scazzocchio B., Varì R., Filesi C., Masella R., Giovannini C. (2008). Modulatory Effects of Polyphenols on Apoptosis Induction: Relevance for Cancer Prevention. Int. J. Mol. Sci..

[9] Mao H., Wen Y., Yu Y., Li H., Wang J., Sun B. (2022). Ignored Role of Polyphenol in Boosting Reactive Oxygen Species Generation for Polyphenol/Chemodynamic Combination Therapy. Mater. Today Bio.

[10] Elena-Real C.A., Díaz-Quintana A., González-Arzola K., Velázquez-Campoy A., Orzáez M., López-Rivas A., Gil-Caballero S., De La Rosa M., Díaz-Moreno I. (2018). Cytochrome c Speeds up Caspase Cascade Activation by Blocking 14-3-3ϵ-Dependent Apaf-1 Inhibition Article. Cell Death Dis..

[11] Nava-Villalba M., Aceves C. (2014). 6-Iodolactone, Key Mediator of Antitumoral Properties of Iodine. Prostaglandins Other Lipid Mediat..

[12] Aranda N., Sosa S., Delgado G., Aceves C., Anguiano B. (2013). Uptake and Antitumoral Effects of Iodine and 6-Iodolactone in Differentiated and Undifferentiated Human Prostate Cancer Cell Lines. Prostate.

[13] Shrivastava A., Tiwari M., Sinha R.A., Kumar A., Balapure A.K., Bajpai V.K., Sharma R., Mitra K., Tandon A., Godbole M.M. (2006). Molecular Iodine Induces Caspase-Independent Apoptosis in Human Breast Carcinoma Cells Involving the Mitochondria-Mediated Pathway. J. Biol. Chem..

[14] Aceves C., García-Solís P., Arroyo-Helguera O., Vega-Riveroll L., Delgado G., Anguiano B. (2009). Antineoplastic Effect of Iodine in Mammary Cancer: Participation of 6-Iodolactone (6-IL) and Peroxisome Proliferator-Activated Receptors (PPAR). Mol. Cancer.

[15] Arroyo-Helguera O., Rojas E., Delgado G., Aceves C. (2008). Signaling Pathways Involved in the Antiproliferative Effect of Molecular Iodine in Normal and Tumoral Breast Cells: Evidence That 6-Iodolactone Mediates Apoptotic Effects. Endocr. Relat. Cancer.

[16] Rösner H., Torremante P., Möller W., Gärtner R. (2009). Antiproliferative/Cytotoxic Activity of Molecular Iodine and Iodolactones in Various Human Carcinoma Cell Lines. No Interfering with EGF-Signaling, but Evidence for Apoptosis. Exp. Clin. Endocrinol. Diabetes.

[17] Sularz O., Smoleń S., Koronowicz A., Kowalska I., Leszczyńska T. (2020). Chemical Composition of Lettuce (*Lactuca sativa* L.) Biofortified with Iodine by KIO3, 5-Iodo-, and 3.5-Diiodosalicylic Acid in a Hydroponic Cultivation. Agronomy.

[18] Sularz O., Koronowicz A., Smoleń S., Kowalska I., Skoczylas Ł., Liszka-Skoczylas M., Tabaszewska M., Pitala J. (2021). Anti- And pro-Oxidant Potential of Lettuce (*Lactuca sativa* L.) Biofortified with Iodine by KIO3, 5-Iodo- And 3,5-Diiodosalicylic Acid in Human Gastrointestinal Cancer Cell Lines. RSC Adv..

[19] Sularz O., Koronowicz A., Boycott C., Smoleń S., Stefanska B. (2022). Molecular Effects of Iodine-Biofortified Lettuce in Human Gastrointestinal Cancer Cells. Nutrients.

[20] Hikisz P., Kiliańska Z.M. (2012). Puma, a Critical Mediator of Cell Death—One Decade on from Its Discovery. Cell. Mol. Biol. Lett..

[21] Pua L.J.W., Mai C.W., Chung F.F.L., Khoo A.S.B., Leong C.O., Lim W.M., Hii L.W. (2022). Functional Roles of JNK and P38 MAPK Signaling in Nasopharyngeal Carcinoma. Int. J. Mol. Sci..

[22] Tomás-Loba A., Manieri E., González-Terán B., Mora A., Leiva-Vega L., Santamans A.M., Romero-Becerra R., Rodríguez E., Pintor-Chocano A., Feixas F. (2019). P38γ Is Essential for Cell Cycle Progression and Liver Tumorigenesis. Nature.

[23] Kisling L.A., Das J.M. (2022). Prevention Strategies. StatPearls.

[24] Rock C.L., Thomson C., Gansler T., Gapstur S.M., McCullough M.L., Patel A.V., Andrews K.S., Bandera E.V., Spees C.K., Robien K. (2020). American Cancer Society Guideline for Diet and Physical Activity for Cancer Prevention. CA Cancer J. Clin..

[25] Zhang Y., Liu K., Yan C., Yin Y., He S., Qiu L., Li G. (2022). Natural Polyphenols for Treatment of Colorectal Cancer. Molecules.

[26] Appunni S., Rubens M., Ramamoorthy V., Tonse R. (2021). Emerging Evidence on the Effects of Dietary Factors on the Gut Microbiome in Colorectal Cancer. Front. Nutr..

[27] Pfeffer C.M., Singh A.T.K. (2018). Apoptosis: A Target for Anticancer Therapy. Int. J. Mol. Sci..

[28] Aceves C., Mendieta I., Anguiano B., Delgado-González E. (2021). Molecular Iodine Has Extrathyroidal Effects as an Antioxidant, Differentiator, and Immunomodulator. Int. J. Mol. Sci..

[29] Nuñez-Anita R.E., Arroyo-Helguera O., Cajero-Juárez M., López-Bojorquez L., Aceves C. (2009). A Complex between 6-Iodolactone and the Peroxisome Proliferator-Activated Receptor Type Gamma May Mediate the Antineoplasic Effect of Iodine in Mammary Cancer. Prostaglandins Other Lipid Mediat..

[30] Mendieta I., Nuñez-Anita R.E., Nava-Villalba M., Zambrano-Estrada X., Delgado-González E., Anguiano B., Aceves C. (2019). Molecular Iodine Exerts Antineoplastic Effects by Diminishing Proliferation and Invasive Potential and Activating the Immune Response in Mammary Cancer Xenografts. BMC Cancer.

[31] Wang D., Ning W., Xie D., Guo L., DuBois R.N. (2012). Peroxisome Proliferator-Activated Receptor δ Confers Resistance to Peroxisome Proliferator-Activated Receptor γ-Induced Apoptosis in Colorectal Cancer Cells. Oncogene.

[32] Nava-Villalba M., Nuñez-Anita R.E., Bontempo A., Aceves C. (2015). Activation of Peroxisome Proliferator-Activated Receptor Gamma Is Crucial for Antitumoral Effects of 6-Iodolactone. Mol. Cancer.

[33] Sanghavi D.M., Thelen M., Thornberry N.A., Casciola-Rosen L., Rosen A. (1998). Caspase-Mediated Proteolysis during Apoptosis: Insights from Apoptotic Neutrophils. FEBS Lett..

[34] Guerrero A.D., Schmitz I., Chen M., Wang J. (2012). Promotion of Caspase Activation by Caspase-9-Mediated Feedback Amplification of Mitochondrial Damage. J. Clin. Cell. Immunol..

[35] Puig B., Tortosa A., Ferrer I. (2001). Cleaved Caspase-3, Caspase-7 and Poly (ADP-Ribose) Polymerase Are Complementarily but Differentially Expressed in Human Medulloblastomas. Neurosci. Lett..

[36] Soriano O., Delgado G., Anguiano B., Petrosyan P., Molina-Servín E.D., Gonsebatt M.E., Aceves C. (2011). Antineoplastic Effect of Iodine and Iodide in Dimethylbenz[a]Anthracene- Induced Mammary Tumors: Association between Lactoperoxidase and Estrogen-Adduct Production. Endocr. Relat. Cancer.

[37] Zhang D., Xu X., Li J., Yang X., Sun J., Wu Y., Qiao H. (2019). High Iodine Effects on the Proliferation, Apoptosis, and Migration of Papillary Thyroid Carcinoma Cells as a Result of Autophagy Induced by BRAF Kinase. Biomed. Pharmacother..

[38] Inoue S., Browne G., Melino G., Cohen G.M. (2009). Ordering of Caspases in Cells Undergoing Apoptosis by the Intrinsic Pathway. Cell Death Differ..

[39] Slee E.A., Adrain C., Martin S.J. (2001). Executioner Caspase-3, -6, and -7 Perform Distinct, Non-Redundant Roles during the Demolition Phase of Apoptosis. J. Biol. Chem..

[40] Bhosale P.B., Ha S.E., Vetrivel P., Kim H.H., Kim S.M., Kim G.S. (2020). Functions of Polyphenols and Its Anticancer Properties in Biomedical Research: A Narrative Review. Transl. Cancer Res..

[41] Duo J., Ying G.G., Wang G.W., Zhang L. (2012). Quercetin Inhibits Human Breast Cancer Cell Proliferation and Induces Apoptosis via Bcl-2 and Bax Regulation. Mol. Med. Rep..

[42] Yang S., Si L., Jia Y., Jian W., Yu Q., Wang M., Lin R. (2019). Kaempferol Exerts Anti-Proliferative Effects on Human Ovarian Cancer Cells by Inducing Apoptosis, G0/G1 Cell Cycle Arrest and Modulation of MEK/ERK and STAT3 Pathways. J. Balk. Union Oncol..

[43] Campbell K.J., Tait S.W.G. (2018). Targeting BCL-2 Regulated Apoptosis in Cancer. Open Biol..

[44] Zhang J., Huang K., O’neill K.L., Pang X., Luo X. (2016). Bax/Bak Activation in the Absence of Bid, Bim, Puma, and P53. Cell Death Dis..

[45] Luo Y., Wu Y., Huang H., Yi N., Chen Y. (2021). Emerging Role of BAD and DAD1 as Potential Targets and Biomarkers in Cancer (Review). Oncol. Lett..

[46] Oltersdorf T., Elmore S.W., Shoemaker A.R., Armstrong R.C., Augeri D.J., Belli B.A., Bruncko M., Deckwerth T.L., Dinges J., Hajduk P.J. (2005). An Inhibitor of Bcl-2 Family Proteins Induces Regression of Solid Tumours. Nature.

[47] Liu B., Yuan B., Zhang L., Mu W., Wang C. (2015). ROS / P38 / P53 / Puma Signaling Pathway Is Involved in Emodin-Induced Apoptosis of Human Colorectal Cancer Cells. Int. J. Clin. Exp. Med..

[48] Song Y., Li X., Li Y., Li N., Shi X., Ding H., Zhang Y., Li X., Liu G., Wang Z. (2014). Non-Esterified Fatty Acids Activate the ROS-P38-P53/Nrf2 Signaling Pathway to Induce Bovine Hepatocyte Apoptosis in Vitro. Apoptosis.

[49] Gräb J., Rybniker J. (2019). The Expanding Role of P38 Mitogen-Activated Protein Kinase in Programmed Host Cell Death. Microbiol. Insights.

[50] Cuenda A., Rousseau S. (2007). P38 MAP-Kinases Pathway Regulation, Function and Role in Human Diseases. Biochim. Biophys. Acta Mol. Cell Res..

[51] Pranteda A., Piastra V., Stramucci L., Fratantonio D., Bossi G. (2020). The P38 Mapk Signaling Activation in Colorectal Cancer upon Therapeutic Treatments. Int. J. Mol. Sci..

[52] Losa J.H., Cobo C.P., Viniegra J.G., Sánchez-Arevalo Lobo V.J., Ramón y Cajal S., Sánchez-Prieto R. (2003). Role of the P38 MAPK Pathway in Cisplatin-Based Therapy. Oncogene.

[53] Liu Y.T., Chuang Y.C., Lo Y.S., Lin C.C., Hsi Y.T., Hsieh M.J., Chen M.K. (2020). Asiatic Acid, Extracted from Centella Asiatica and Induces Apoptosis Pathway through the Phosphorylation P38 Mitogen-Activated Protein Kinase in Cisplatin-Resistant Nasopharyngeal Carcinoma Cells. Biomolecules.

[54] Hiraishi N., Kanmura S., Oda K., Arima S., Kumagai K., Mawatari S., Tanoue S., Sasaki F., Hashimoto S., Ido A. (2019). Extract of Lactobacillus Plantarum Strain 06CC2 Induces JNK/P38 MAPK Pathway-Mediated Apoptosis through Endoplasmic Reticulum Stress in Caco_2_ Colorectal Cancer Cells. Biochem. Biophys. Rep..

[55] Whitaker R.H., Cook J.G. (2021). Stress Relief Techniques: P38 MAPK Determines the Balance of Cell Cycle and Apoptosis Pathways. Biomolecules.

[56] Zhuang S., Demirs J.T., Kochevar I.E. (2000). P38 Mitogen-Activated Protein Kinase Mediates Bid Cleavage, Mitochondrial Dysfunction, and Caspase-3 Activation During Apoptosis Induced By Singlet Oxygen But Not By Hydrogen Peroxide. J. Biol. Chem..

[57] Pereira L., Igea A., Canovas B., Dolado I., Nebreda A.R. (2013). Inhibition of P38 MAPK Sensitizes Tumour Cells to Cisplatin-Induced Apoptosis Mediated by Reactive Oxygen Species and JNK. EMBO Mol. Med..

[58] Sudo T., Maruyama M., Osada H. (2002). Stress Meets Development in P38 MAP Kinase. Prog. Biotechnol..

[59] Rouse J., Cohen P., Trigon S., Morange M., Alonso-Llamazares A., Zamanillo D., Hunt T., Nebreda A.R. (1994). A Novel Kinase Cascade Triggered by Stress and Heat Shock That Stimulates MAPKAP Kinase-2 and Phosphorylation of the Small Heat Shock Proteins. Cell.

[60] Zheng C., Lin Z., Zhao Z.J., Yang Y., Niu H., Shen X. (2006). MAPK-Activated Protein Kinase-2 (MK2)-Mediated Formation and Phosphorylation-Regulated Dissociation of the Signal Complex Consisting of P38, MK2, Akt, and Hsp27. J. Biol. Chem..

[61] Ricci J.E., Maulon L., Battaglione-Hofman V., Bertolotto C., Luciano F., Mari B., Hofman P., Auberger P. (2001). A Jurkat T Cell Variant Resistant to Death Receptor-Induced Apoptosis. Correlation with Heat Shock Protein (Hsp) 27 and 70 Levels. Eur. Cytokine Netw..

[62] Seul-Ki C., Kam H., Kye-Young K., In Park S., Yun-Sil L. (2019). Targeting Heat Shock Protein 27 in Cancer: A Druggable Target for Cancer Treatment?. Cancers.

[63] Concannon C.G., Orrenius S., Samali A. (2001). Hsp27 Inhibits Cytochrome C-Mediated Caspase Activation by Sequestering Both pro-Caspase-3 and Cytochrome c. Gene Expr..

[64] Umar H.I., Ajayi A.T., Mukerjee N., Aborode A.T., Hasan M.M., Maitra S., Bello R.O., Alabere H.O., Sanusi A.A., Awolaja O.O. (2022). Discovery of Novel HSP27 Inhibitors as Prospective Anti-Cancer Agents Utilizing Computer-Assisted Therapeutic Discovery Approaches. Cells.

[65] Zhu C., Jiang H., Deng W., Zhao S., Li K., Wang Y., Wei Q., Du J. (2019). Activation of P38/HSP27 Pathway Counters Melatonin-Induced Inhibitory Effect on Proliferation of Human Gastric Cancer Cells. J. Biomed. Res..

[66] Li X., Miao X., Wang H., Xu Z., Li B. (2015). The Tissue Dependent Interactions between P53 and Bcl-2 in Vivo. Oncotarget.

[67] Chipuk J.E., Green D.R. (2006). Dissecting P53-Dependent Apoptosis. Cell Death Differ..

[68] Roufayel R., Younes K., Al-Sabi A., Murshid N. (2022). BH3-Only Proteins Noxa and Puma Are Key Regulators of Induced Apoptosis. Life.

[69] Kondratovskii P.M., Dubikov A.I., Doroshevskaya A.Y., Eliseikina M.G. (2014). PUMA Protein in P53 Regulatory Molecule Pattern Determines the Prognosis for Patients with Lymphoproliferative Diseases. Bull. Exp. Biol. Med..

[70] Ray R.M., Bhattacharya S., Johnson L.R. (2011). Mdm2 Inhibition Induces Apoptosis in P53 Deficient Human Colon Cancer Cells by Activating P73- and E2F1-Mediated Expression of PUMA and Siva-1. Apoptosis.

[71] Faruq F., Zhao D., Wu J., George William J.N., Zhang M., Chang H. (2019). Downregulation of MDM2 Leads to Anti-Proliferative Effects through Activation of P53-Associated Pathway Mediated By Both Dual Inhibitor MX69 and Mir-548c-3p in Multiple Myeloma. Blood.

[72] Taylor C.A., Zheng Q., Liu Z., Thompson J.E. (2013). Role of P38 and JNK MAPK Signaling Pathways and Tumor Suppressor P53 on Induction of Apoptosis in Response to Ad-EIF5A1 in A549 Lung Cancer Cells. Mol. Cancer.

[73] Behrens A., Sibilia M., Wagner E.F. (1999). Amino-Terminal Phosphorylation of c-Jun Regulates Stress-Induced Apoptosis and Cellular Proliferation. Nat. Genet..

[74] Brennan A., Leech J.T., Kad N.M., Mason J.M. (2020). Selective Antagonism of CJun for Cancer Therapy. J. Exp. Clin. Cancer Res..

[75] Podar K., Raab M.S., Tonon G., Sattler M., Barilà D., Zhang J., Tai Y.T., Yasui H., Raje N., DePinho R.A. (2007). Up-Regulation of c-Jun Inhibits Proliferation and Induces Apoptosis via Caspase-Triggered c-Abl Cleavage in Human Multiple Myeloma. Cancer Res..

[76] Wang N., Verna L., Hardy S., Zhu Y., Ma K.S., Birrer M.J., Stemerman M.B. (1997). C-Jun Triggers Apoptosis in Human Vascular Endothelial Cells. Circ. Res..

[77] Humar M., Loop T., Schmidt R., Hoetzel A., Roesslein M., Andriopoulos N., Pahl H.L., Geiger K.K., Pannen B.H.J. (2007). The Mitogen-Activated Protein Kinase P38 Regulates Activator Protein 1 by Direct Phosphorylation of c-Jun. Int. J. Biochem. Cell Biol..

[78] Sánchez-Pérez I., Perona R. (1999). Lack of C-Jun Activity Increases Survival to Cisplatin. FEBS Lett..

