# Supplementary material for: Iodine-Biofortified Lettuce Can Promote Mitochondrial Dependent Pathway of Apoptosis in Human Gastrointestinal Cancer Cells
Source: Int J Mol Sci. 2023 Jun 7;24(12):9869. doi: 10.3390/ijms24129869 (PMC10298746; doi:10.3390/ijms24129869)
Supplement: Supplementary file 1 [file ijms-24-09869-s001.zip › ijms-2418629-supplementary.pdf]

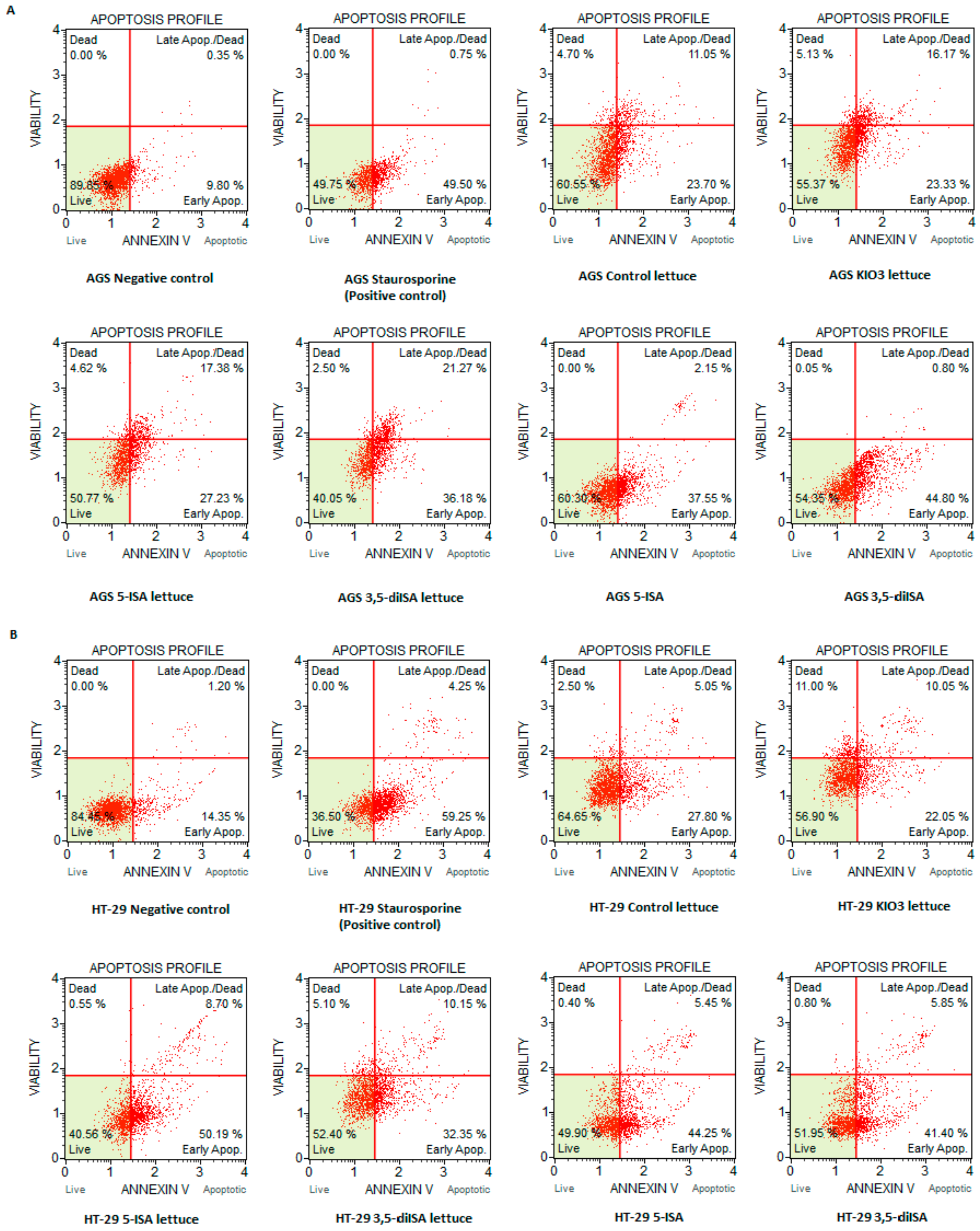

Supplementary Figure S1. Representative plots from Muse® Annexin V & Dead Cell Assay. The effect of extracts from iodine-biofortified lettuce on apoptosis in human gastrointestinal cell line AGS (A) and colorectal adenocarcinoma cell line HT-29 (B). The above dot plots show the histograms presenting four cell populations – live, early apoptotic, late apoptotic, and dead cells.

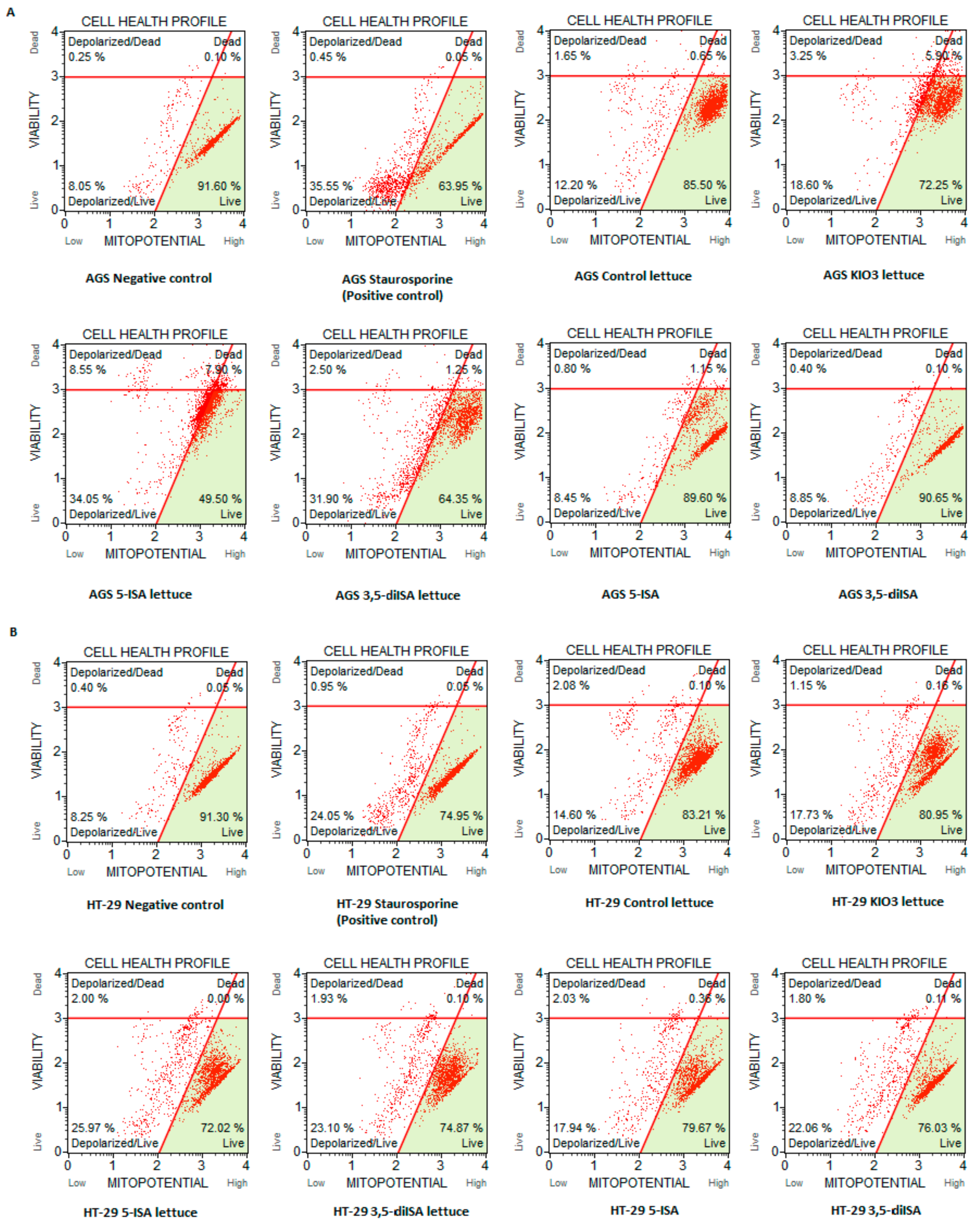

Supplementary Figure S2. Representative plots from Muse® MitoPotential Assay. The effect of extracts from iodine-biofortified lettuce on mitochondrial membrane potential in human gastrointestinal cell line AGS (A) and colorectal adenocarcinoma cell line HT-29 (B). The above dot plots show the histograms presenting four cell populations – live, live/depolarized, dead/depolarized, and dead cells.
